# Comparative Analysis of Controlled Ovarian Hyperstimulation and Modified Natural Cycle Protocols on Gene Expression and Quality of Oocytes, Zygotes, and Embryos in Assisted Reproductive Technology (ART)

**DOI:** 10.3390/ijms252413287

**Published:** 2024-12-11

**Authors:** Sanja Dević Pavlić, Lara Saftić Martinović, Tina Sušanj Šepić, Anđelka Radojčić Badovinac

**Affiliations:** 1 Department of Medical Biology and Genetics, Faculty of Medicine, University of Rijeka, HR-51000 Rijeka, Croatia; lara.saftic.martinovic@medri.uniri.hr; 2 Clinic for Gynecology and Obstetrics, Clinical Hospital Center Rijeka, HR-51000 Rijeka, Croatia; tina.susanj@gmail.com; 3 Faculty of Biotechnology and Drug Development, University of Rijeka, HR-51000 Rijeka, Croatia; andjelka@uniri.hr

**Keywords:** assisted reproductive technology, controlled ovarian hyperstimulation, in vitro fertilization, gene expression, oocyte grade

## Abstract

This study investigated the influence of two in vitro fertilization (IVF) protocols—controlled ovarian hyperstimulation (COH) and a modified natural cycle protocol—on gene expression levels (Anti-Müllerian Hormone (*AMH*), Anti-Müllerian Hormone Receptor Type 2 (*AMHAMHR2*), Follicle-Stimulating Hormone Receptor (*FSHR*), and Androgen Receptor (*AR*)) and the subsequent reproductive outcomes of assisted reproductive technology (ART). Gene expression, as well as oocyte, zygote, and embryo morphological parameters, were analyzed to evaluate the differences between the protocols. Our findings show that *AMH* expression was significantly associated with successful fertilization, while *AMHAMHR2* expression correlated with improved embryo transfer outcomes. The modified natural cycle protocol demonstrated a higher association with the favorable gene expression profiles, particularly for *AMH* and *AMHAMHR2*, linked to successful fertilization and embryo transfer, suggesting potential advantages of minimal intervention. However, the overall quality scores for the oocytes, zygotes, and embryos were comparable between the protocols. The trend of a higher transfer success for the natural cycle, though not statistically significant, indicated potential protocol effects on the uterine environment. This study highlights the complexity of ART outcomes and suggests that incorporating gene expression markers with protocol adjustments may optimize individual ART strategies.

## 1. Introduction

Assisted reproductive technology (ART) refers to medical procedures primarily used to treat infertility, such as intracytoplasmic sperm injection (ICSI) and other methods, which aid in the conception and development of embryos outside the human body. The success of ART is heavily dependent on the quality of the oocytes, zygotes, and embryos, which can be influenced by a variety of factors, including ovarian stimulation protocols and gene expression profiles.

The oocyte microenvironment, primarily composed of cumulus cells forming the cumulus–oocyte complex (COC), is crucial for the growth and development of an oocyte [1]. The proper development of an oocyte capable of completing meiosis, fertilization, and the formation of an embryo depends upon the intricate signaling and communication between the oocyte itself and its surrounding cells, and requires the proper development of the follicle. During folliculogenesis, the oocyte interacts with undifferentiated granulosa cells, which later differentiate into cumulus cells [2,3]. These cumulus cells provide the necessary support for oocyte development, although the understanding of their interactions is still incomplete [4]. Bidirectional communication between the cumulus cells and the oocyte is essential for their coordinated growth and development [5].

Recent research has increasingly focused on the molecules involved in cellular signaling and metabolism within the oocyte microenvironment. One of these molecules, Anti-Müllerian Hormone (*AMH*), which is expressed in the cumulus cells, plays a crucial role in folliculogenesis and the maturation of oocytes. AMH, a glycoprotein growth factor, is involved in cell growth, extracellular matrix production, tissue remodeling, and interactions between the embryonic connective tissue and epithelium [6]. Secreted exclusively by granulosa cells from the 36th gestational week until menopause, AMH exhibits specific expression patterns during folliculogenesis, underscoring its role in regulating follicle growth and selection for ovulation. Studies by Durlinger et al. have demonstrated that the absence of AMH increases the sensitivity of follicles to FSH, thereby inhibiting the initial recruitment of primordial follicles and affecting FSH-dependent follicle growth. This regulation affects the number of growing follicles and can impair the quality of oocytes [7,8,9,10].

**Figure 1:**
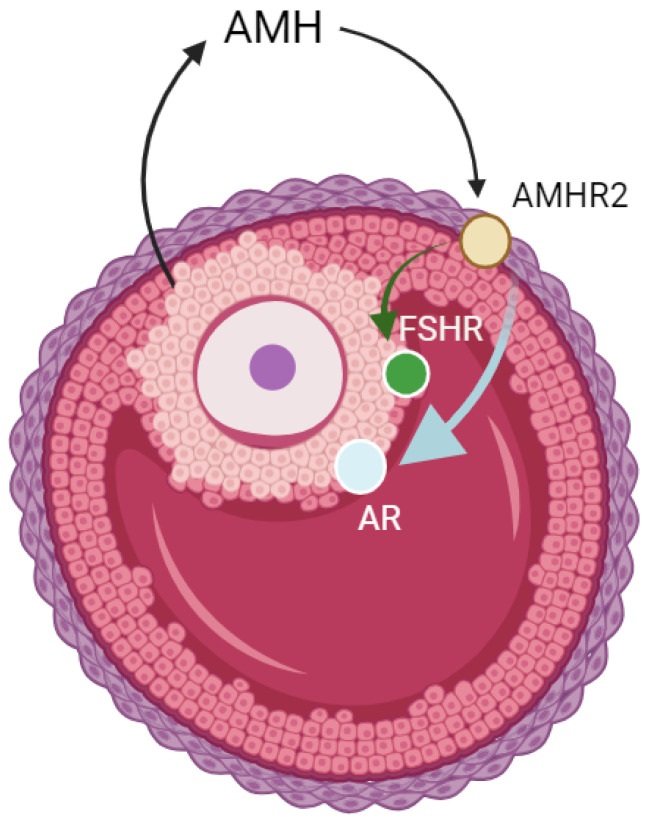
Anti-Müllerian Hormone (*AMH*), signaling pathway in granulosa cells. This figure shows AMH binding to its receptor Anti-Müllerian Hormone Receptor Type 2 (*AMHR2*) on granulosa cells, regulating folliculogenesis. Follicle-Stimulating Hormone Receptor (*FSHR*), and Androgen Receptor (*AR*) are also depicted, reflecting their roles in follicular development and hormone response. AMH modulates follicle recruitment and Follicle-Stimulating Hormone (*FSH*) sensitivity, essential for ovarian reserve and fertility. Created with BioRender.com.

As with other members of the TGF-β superfamily, AMH signaling is mediated by a serine–threonine kinase receptor complex. This complex comprises ligand-specific type II receptors and broader-acting type I receptors. The type II receptor (AMHR2) is essential for AMH signaling, as has been shown in studies of *AMHAMHR2*-deficient mice [11]. The expression of *AMH* in the cumulus cells correlates significantly with its concentration in the follicular fluid, and is positively associated with the expression of *AMHAMHR2*, *FSHR*, and *AR* [12,13]. Additionally, the expression of *AMH* and *AR* genes in cumulus cells has been associated with oocyte quality [14]. An overview of these genes and their roles in ovarian function are summarized in Table 1 and Figure 1.

The quality of the oocytes is a crucial factor for the success of ART, and the most common method for assessing their quality is to evaluate their morphology. The morphological characterization of the oocytes provides information about their developmental potential and is typically used for the selection of oocytes for ART. However, this method is not always accurate for predicting fertilization success and zygote developmental capacity [17,18]. In addition to the assessment of oocyte morphology, assessing the morphology of the zygote and the resulting embryo after fertilization is crucial for determining fertilization success and selecting the embryos for transfer.

Controlled ovarian hyperstimulation (COH), a common procedure for women undergoing ART, often results in oocytes of variable quality, due to the uneven maturation of their nuclei and cytoplasms [19]. By elevating gonadotropins, COH influences the transcription of the genes involved in cell cycle regulation, mitochondrial function, and stress responses. For instance, Grøndahl et al. explored the effects of COH using recombinant FSH and urinary hMG, demonstrating distinct gene expression patterns in granulosa cells, where the genes linked to steroid synthesis and anti-apoptotic functions were differentially expressed depending on the stimulation method [20]. This highlights the fact that COH protocols can distinctly modulate the genes critical for follicular development and oocyte quality. Similarly, Liu et al. focused on COH’s impact on connexin43 (Cx43) in endometrial stromal cells, finding that the high estrogen levels from COH protocols suppressed Cx43 expression [21]. Mirkin et al. investigated gene expression changes in the peri-implantation endometrium during COH cycles compared to natural cycles, identifying gene downregulation associated with endometrial receptivity. This suggests that the hormonal changes induced by COH could alter gene regulation in a way that impacts implantation and possibly broader follicular functions [22]. Another research study showed that cumulus cells in COH exhibit an increased expression of genes, such as CYP19A1, linked to steroid synthesis, and stress-related miRNAs like miR-21, which can introduce oxidative stress and disrupt normal oocyte maturation. These gene expression changes compromise oocyte quality and may affect fertilization and embryo viability, highlighting the importance of carefully tailored COH protocols [23,24]. Collectively, these findings underscore the need for further analysis of gene expression under varying COH protocols to better understand their influence on the genes critical to folliculogenesis and reproductive outcomes.

The purpose of this study was to evaluate the impact of COH—specifically the antagonistic hormonal stimulation protocol (A) and the modified natural cycle (N)—on the various outcomes of ART. This included assessing the gene expression levels of *AMH*, *AMHAMHR2*, *FSHR*, and *AR*, as well as evaluating the quality of oocytes, zygotes, and embryos. Additionally, this study aimed to determine whether these protocols influenced fertilization rates, successful fertilization rates, and embryo transfer success rates. By comparing these results, this study aimed to better understand the effectiveness of the COH procedure, with the intention of contributing to the refinement of ART treatment approaches and supporting improvements in reproductive medicine practices.

## 2. Results and Discussion

This study aimed to explore whether the two IVF protocols differentially influenced the gene expressions of *AMH*, *AMHAMHR2*, *FSHR*, and *AR* and, subsequently, affected the morphological grades of the oocytes, zygotes, and embryos. Understanding the relationship between these protocols and gene expression is vital, as these genes are integral to follicular development, ovarian reserve, and overall reproductive cell quality.

### 2.1. Comparison of Gene Expression Levels Between Antagonistic COH and Modified Natural Cycle Protocols

**Figure 2:**
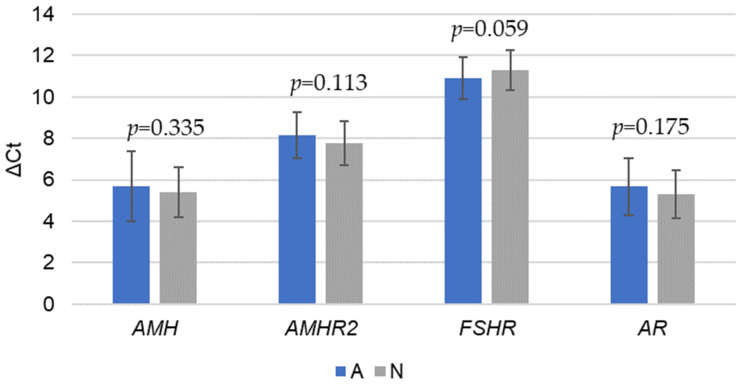
Comparison of gene expression levels (ΔCt values) between antagonistic (A) COH and modified natural cycle (N) IVF protocols (significant difference, *p* < 0.05).

The statistical analysis conducted on the gene expression levels for *AMH*, *AMHAMHR2*, *FSHR*, and *AR* between the antagonistic and natural cycle protocols indicated no statistically significant differences (*p*-values: 0.335, 0.113, 0.059, and 0.175, respectively) (Figure 2).

However, the results show that the mean *AMH* expression was slightly higher in the A group (5.70 ± 1.68) compared to the N group (5.40 ± 1.21), suggesting a marginal increase in *AMH* expression with the antagonistic protocol. In addition, the mean *AMHAMHR2* expression was higher in the A group (8.14 ± 1.11), indicating that the antagonistic protocol may lead to an increased expression of this gene, which is moderately correlated with better oocyte and embryo quality. In the N protocol, *FSHR* gene expression was notably higher. This near-significant difference (*p* = 0.059) suggests that *FSHR* expression may be more pronounced when an ovarian cycle is allowed to progress without pharmacological suppression. The GnRH antagonists’ hormonal intervention may suppress the natural upregulation of *FSHR*, leading to comparatively lower expression levels. This difference may reflect a regulatory mechanism wherein the natural cycle allows for a higher sensitivity to endogenous FSH, possibly supporting follicular growth more consistently with the body’s natural hormonal rhythms.

### 2.2. Comparative Analysis of Oocyte, Zygote, and Embryo Morphology Between Antagonistic COH and Modified Natural Cycle Protocols

To determine if there were statistically significant differences between the grades of the oocytes, zygotes, and embryos (defined in Table 2) based on the type of IVF protocol used (A vs. N), chi-square tests were performed on the contingency tables for each grade (Table 3). The grades for the oocytes, zygotes, and embryos did not show statistically significant differences between the two protocols (*p*-values: 0.607, 0.592, and 0.600, respectively). These results indicate that both protocols produced a comparable quality of reproductive cells, with no protocol showing superior efficacy at improving the cell grades.

For the oocyte grades, the antagonistic protocol group had a mean grade of 1.44 ± 0.75, whereas the modified natural cycle protocol group had a slightly higher mean grade of 1.59 ± 0.86. This suggests that both protocols produce similar oocyte quality, with the natural cycle protocol showing slightly more variability. In terms of the zygote grades, the A group had a mean grade of 1.73 ± 0.86, while the N group had a mean grade of 1.91 ± 0.90. Again, the grades are comparable, with the natural cycle protocol having a slightly higher average grade and variability. In terms of the embryonic grades, the A group had a mean grade of 2.34 ± 0.81, while the N group had a mean grade of 2.18 ± 0.85. This indicates that the embryos produced by the antagonistic protocol have slightly higher grades on average, though the difference is small, and both protocols exhibit similar variability.

Furthermore, we contrasted specific oocyte and zygote morphological parameters between the A COH and N protocols. These parameters included the presence of a large perivitelline space (PVS), which indicates abnormal oocyte maturation; inclusions within the PVS, reflecting cytoplasmic dysmaturity; and the size of the first polar body (1. PB), indicating chromosome segregation issues. We also examined the accumulation of organelles in the cytoplasm, affecting oocyte metabolism; smooth endoplasmic reticulum (SER) disc structures, linked to lipid and protein synthesis; and large or many small vacuoles, indicating cytoplasmic immaturity. These parameters collectively determine oocyte maturation, cytoplasmic integrity, and developmental competence, which are crucial for successful fertilization and embryonic development. To ascertain whether there were statistically significant differences between the two groups for each parameter, an analysis was carried out using a *t*-test (Table 4). Our analysis shows that none of the specific parameters examined were affected by the type of IVF protocol. The fact that these parameters did not significantly differ between the A and N protocols implies that the specific factors affecting oocyte quality are not affected by the hormonal protocol selection.

In addition, we also analyzed four zygote quality parameters to investigate whether the hormonal stimulation technique affected the specific zygote parameters (despite the fact that we previously demonstrated that it did not change the zygote’s total score) (Table 5). These parameters included differences in the PN sizes, indicating developmental abnormalities; asymmetry in the PN positions (not positioned in the middle of the cytoplasm), indicating cytoplasmic or nuclear anomalies; and the number and position of nucleolar precursor bodies (NPBs), reflecting issues in nucleolar formation. The presence of widely separated PN indicating asynchronous DNA replication was not included in the analysis because the mean value for both groups (A and N) was consistently 0.00 ± 0.00, indicating no variation or meaningful data for comparison. These characteristics are critical for determining zygote viability and the likelihood of successful embryo development because they reflect the early phases of fertilization and chromosomal integrity. The analysis revealed no statistically significant differences in the zygote quality parameters between the antagonistic hormonal stimulation protocol and the natural cycle.

### 2.3. Comparative Analysis of Gene Expression Levels on Oocyte and Zygote Quality Parameters

Lastly, we analyzed the effects of *AMH*, *AMHAMHR2*, *FSHR*, and *AR* gene expression levels on the oocyte and zygote quality parameters regardless of the IVF protocol used (Table 6 and Table 7).

The results show no correlations between any of the analyzed gene expression levels and the individual morphological oocyte quality parameters (Table 6). While *AMH*, *AMHAMHR2*, *FSHR*, and *AR* are recognized as essential for follicular development, their roles in influencing specific morphological features of fully matured oocytes may be indirect. For instance, *AMH* primarily modulates follicle recruitment and sensitivity to FSH, but its direct impact on oocyte cytoplasmic or extracytoplasmic features remains less clear. This may explain why *AMH* showed only weak correlations with parameters such as PVS inclusions and organelle accumulation. In the literature, studies have frequently assessed the cumulus cells surrounding an oocyte, rather than the oocyte itself, when exploring the correlations between gene expression and oocyte quality. Montazeri et. al. showed that *FSHR* and *AMHAMHR2* are particularly upregulated in cumulus cells during specific maturation stages, suggesting that cumulus cells might serve as a more effective proxy for oocyte developmental potential [25]. In conclusion, while our study involved oocytes at the same maturation stage, the weak correlations for most of the analyzed genes expression levels we observed may stem from interindividual variability, the indirect role of these genes on post-maturation morphological features, and the limitations of assessing gene expression directly in oocytes rather than in the supportive cumulus cells. This highlights the potential value of combining oocyte morphology with cumulus cell gene expression profiles to better predict oocyte quality and improve assisted reproductive outcomes.

Furthermore, the multivariate regression analysis revealed weak associations between the gene expression levels and zygote quality parameters, which is consistent with some published findings, but highlights the complexity of genetic influences on early embryo development (Table 7). For instance, *AMH* and *AMHAMHR2* expression have been shown to influence follicular development and oocyte competence, particularly through their roles in cumulus and granulosa cells, but direct associations with specific zygote features, such as pronuclear positioning or size variation, remain less definitive. Studies have demonstrated that gene expression in the surrounding cells, like cumulus cells, can serve as markers for oocyte and embryo quality, yet the relationships are often modest, reflecting the multifaceted regulation of early embryogenesis [26]. Moreover, the research on granulosa and cumulus cells has suggested that gene expressions, like that of the luteinizing hormone receptor (*LHR*), can predict oocyte quality in certain IVF populations, particularly in poor responders, but these markers tend to have a stronger association with broader developmental competence rather than the precise morphological characteristics of zygotes [27,28]. These findings support the idea that while gene expression influences early reproductive stages, its role in determining specific zygote morphology parameters is likely limited.

In addition, it has been shown that the use of GnRH agonists or antagonists does not significantly alter the gene expression of *AMHAMHR2* and *FSHR* in mature MII oocytes. This finding aligns with our results, supporting the comparable effectiveness of both the antagonistic and natural protocols in terms of oocyte maturation. Devjak et al. further demonstrated that gene expression in CCs, particularly for *AMHAMHR2* and *FSHR*, remains consistent across the different stimulation approaches, underscoring the adaptability of these protocols in ART, without compromising oocyte quality [23].

Overall, these results demonstrate weak associations between gene expression levels and oocyte/zygote quality. This likely reflects the multifactorial nature of reproductive outcomes, where multiple genetic, environmental, and physiological factors contribute to oocyte and embryo development. Moreover, the limited strength of these relationships may be due to the relatively small sample size and the focus on only a subset of genes, which may not capture the full complexity of gene–oocyte and gene–zygote interactions. Future studies should aim to incorporate a broader set of genetic markers and larger cohorts to better understand the role of gene expression in determining reproductive quality.

Moreover, we conducted a multivariate regression analysis of the gene expression levels between the same oocyte and zygote parameters, but taking into consideration the different IVF protocols (Tables S1–S4). The results also confirm the previously shown weak correlations between *AMH*, *AMHAMHR2*, *FSHR*, and *AR* expression levels and oocyte and zygote quality parameters, regardless of the procedure used (A or N).

### 2.4. Influence of Gene Expression Levels and COH Protocol on Fertilization and Embryo Transfer Success

The final analysis included the evaluation of the influence of A and N IVF protocols on the ART outcomes, such as fertilization and embryo transfer success (Table 8). There was no significant difference in the fertilization success rates between the two protocols, with 67.5% of the fertilized oocytes in the antagonistic protocol group and 73.1% of those in the natural cycle group progressing successfully. This demonstrates that, once fertilized, the chances of developing into a viable embryo are comparable between the two methods. The embryo transfer success rate results indicate that the natural cycle protocol achieved a higher success rate (61.5%) compared to the antagonistic protocol (46.2%). Although this trend suggests that the natural cycle may support better implantation outcomes, the difference between the protocols did not reach statistical significance (*p* = 0.335). This lack of significance implies that both protocols yielded comparable results for embryo transfer success in this dataset. However, the observed trend toward higher success for the natural cycle could still be meaningful clinically, hinting at a potentially favorable alignment between the embryo and uterine environments in this protocol. Further studies with larger sample sizes may help clarify whether this difference could become statistically significant, offering deeper insights into optimizing ART outcomes. The increased success rate for the natural cycle may be due to the more biologically synchronized hormonal environment, which more closely mirrors the body’s natural rhythms, potentially enhancing endometrial receptivity [29]. In contrast, the use of pharmacological agents in the antagonistic protocol may disrupt this balance, negatively affecting the uterine environment and endometrial receptivity, thus lowering the chances of successful implantation. These findings underscore the importance of the uterine environment and hormonal regulation during embryo transfer, highlighting that while fertilization success may not differ between the protocols, the conditions that follow—particularly those governing implantation—may be significantly influenced by the type of ovarian stimulation protocol used. Consequently, the natural cycle may offer a distinct advantage for facilitating successful embryo implantation, making it a favorable option for certain patient populations using ART.

**Figure 3:**
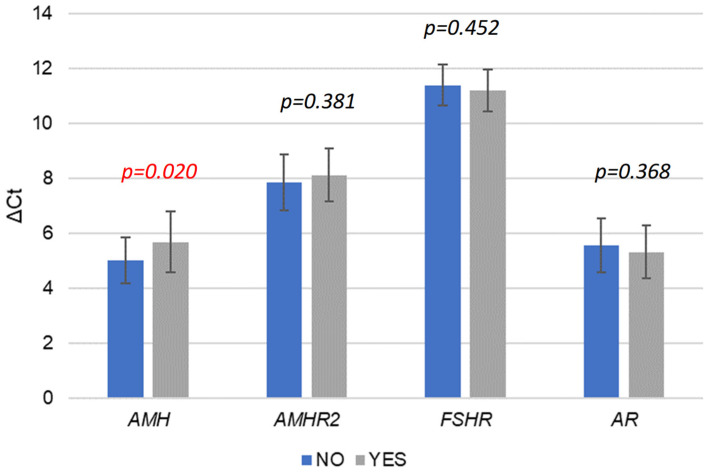
Comparison of gene expression levels (ΔCt values) between fertilization success outcome (successful “YES” vs. unsuccessful “NO”). Significant difference, *p* < 0.05 (in red).

Furthermore, we analyzed whether different gene expression levels influenced the same ART outcomes (fertilization and embryo transfer success). Figure 3 shows the influence of gene expression levels on fertilization success. The results indicate that *AMH* expression is significantly higher in cases with successful fertilization (*p* = 0.020), suggesting that elevated *AMH* may positively impact fertilization outcomes. This finding aligns with *AMH*’s known role in supporting follicle development and oocyte quality, potentially creating a more favorable environment for fertilization [6]. In contrast, the expression levels of *AMHAMHR2* (*p* = 0.381), *FSHR* (*p* = 0.452), and *AR* (*p* = 0.368) did not differ significantly between successful and unsuccessful fertilization outcomes. This lack of association implies that while these genes are critical for broader ovarian and follicular processes, they may not directly influence the immediate success of fertilization. These results highlight *AMH* as a potentially valuable marker for fertilization success, whereas *AMHAMHR2*, *FSHR*, and *AR* may have a more indirect role in this specific outcome.

**Figure 4:**
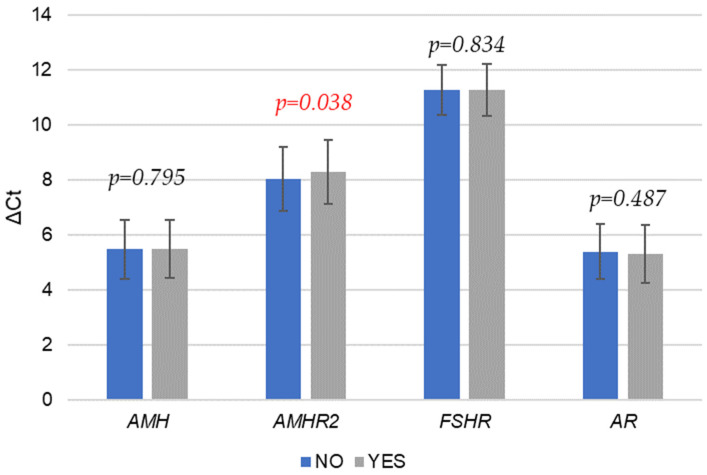
Comparison of gene expression levels (ΔCt values) between embryo transfer success outcome (successful “YES” vs. unsuccessful “NO”). Significant difference, *p* < 0.05 (in red).

The results presented in Figure 4 demonstrate the influence of gene expression levels on embryo transfer success, indicating a significant association between a higher *AMHAMHR2* expression and successful embryo transfer (*p* = 0.038). This suggests that *AMHAMHR2*, which is integral to FSH signaling, may contribute positively to the uterine or follicular environment, enhancing the conditions for successful implantation. The importance of *AMHAMHR2* may be linked to its influence on follicular maturation or endometrial receptivity, both critical for embryo implantation [30]. In contrast, *AMH* expression shows no significant difference between successful and unsuccessful embryo transfers (*p* = 0.795), suggesting that, while *AMH* is essential for oocyte quality and follicle development, its levels may not directly impact transfer success in the final stages of implantation. Similarly, *FSHR* expression, representing the FSH receptor’s influence, does not differ significantly between the groups (*p* = 0.834), indicating that while *FSHR* is crucial for follicular responsiveness, its role may not extend to the specific context of embryo transfer. Finally, *AR* expression also shows no significant difference (*p* = 0.487), implying that Androgen Receptor levels may not have a direct impact on implantation success. Collectively, these findings highlight *AMHAMHR2* as a potential marker for successful embryo transfer, whereas *AMH*, *FSHR*, and *AR* appear to play foundational roles in follicle development rather than influencing the final transfer outcome.

Finally, we analyzed the influence of gene expression levels on the fertilization and embryo transfer success under the A COH protocol and modified natural cycle protocol (Figures S1–S4). The results reveal that the gene expression levels varied between the antagonistic COH and modified natural cycle protocols, impacting the ART outcomes differently. Under the antagonistic COH protocol, higher *AMH* values were observed in the group with successful fertilization (Figure S1). However, there were no statistical significantly changed levels of expression of the *AMH*, *AMHAMHR2*, *FSHR*, and *AR* genes in terms of successful embryo transfer (Figure S3). In addition, the results show no statistically significant differences in the influence of gene expression levels on successful fertilization (Figure S2) or embryo transfer (Figure S4) under the modified natural cycle protocol.

Although there are slight differences between the A COH and modified natural cycle protocols, the similar performance of both protocols demonstrates the robustness of current ovarian stimulation approaches in ART. This underlines the necessity of focusing on other areas of therapy personalization, such as monitoring patient-specific reactions and adapting protocols as needed, rather than rigorously following one protocol over another. A retrospective cohort study of 4402 patients compared the cumulative live birth rates (CLBRs) between flexible GnRH antagonist and standard GnRH agonist protocols, adjusting for factors like age, BMI, and ovarian reserve. Overall, the CLBRs were similar across the protocols; however, younger patients with a high ovarian reserve showed slightly improved outcomes with the GnRH antagonist, indicating a potential benefit for this subgroup without compromising the success rates [31]. Another large-scale cohort analysis supported these findings, revealing that while the stimulation time and peak estradiol levels varied amongst the procedures, they had no effect on embryo quality or cumulative pregnancy rates. Thus, both treatments achieved equivalent CLBRs, indicating flexibility in protocol selection based on unique patient characteristics rather than efficacy alone [32].

### 2.5. Future Perspectives

This study’s strengths lie in its detailed comparison of the antagonistic COH and modified natural cycle IVF protocols, utilizing a gene expression analysis alongside comprehensive morphological assessments of oocytes, zygotes, and embryos. While the results indicate that the natural cycle protocol does not consistently outperform the antagonistic protocol, the findings highlight the potential influence of gene expression levels on ART outcomes.

However, increasing the sample size in future studies would enhance the generalizability and impact of these findings. The small sample size in this study represents a notable limitation, reducing the statistical power to detect more nuanced differences between the protocols. Expanding the scope of the biomarkers for oocyte competence, beyond primary markers like *AMHAMHR2* and *FSHR*, may enhance oocyte quality assessments. Additional genes, such as *SERPINE2* and *VEGFC*, have shown a potential influence on oocyte and follicle development [23]. *SERPINE2* expression, which is regulated by FSH, plays a role in modulating the gene profile within the follicular fluid, potentially affecting the follicular environment and oocyte quality. *VEGFC*, essential for vascularization, has a recognized function in follicular development, with its elevated expression correlating to higher-quality oocytes. Including these genes as part of a multi-marker biomarker approach could refine oocyte selection strategies, contributing to more targeted and effective assisted reproductive technologies.

Pregnancy was not included as an outcome in this study, given the considerable influence of external factors beyond that of the controlled variables. Factors, such as partner gamete quality, lifestyle, stress, and time attempting conception, introduce variability, complicating a precise assessment. Additionally, the low pregnancy rate among the participants limits the statistical power for meaningful conclusions. It is essential to note that the male gamete contribution significantly impacts pregnancy outcomes, presenting another limitation to isolating female-specific factors. By focusing on fertilization and the embryo transfer stages, this study provides a more controlled assessment of female-specific markers within COH protocols, laying a foundation for more targeted approaches to ART.

## 3. Materials and Methods

### 3.1. Subjects

A total of 124 COCs were included in this study, retrieved from 55 patients undergoing assisted reproduction by ICSI at the Department for Human Reproduction at the Clinic for Gynecology and Obstetrics, Clinical Hospital Centre Rijeka, Croatia. All of the study participants were healthy, normo-ovulatory women divided in two groups based on the applied protocol: 32 patients underwent a modified natural cycle and 23 patients underwent COH. The decision on the type of protocol was made individually and in agreement with the patients. All the patients had a BMI within a range of 18 to 24 kg/m^2^. The average age of the patients in the COH group (A) was 35.6 years, while the average age of those in the modified natural cycle group (N) was 34.8 years (*p* = 0.276). From the patients who underwent a modified natural cycle procedure, one or more oocytes were collected from the ovaries during a spontaneous menstrual cycle, with the administration of human chorionic gonadotrophin (hCG) to induce final oocyte maturation [33,34]. Serial ultrasound examinations were used to monitor follicular and endometrial growth starting on day 8. When the leading follicle had a diameter of 17 mm, recombinant chorionic gonadotropin was administered (Ovitrelle^®^, 250 µg choriogonadotropin alfa, Merck Serono, Darmstadt, Germany). After 34–36 h, follicular aspiration (Cook’s aspiration needle) was performed under sonographic guidance. A flexible antagonistic stimulation protocol (A) was used for COH, starting on day two of the menstrual cycle. Recombinant FSH was injected daily to stimulate the ovaries (Gonal-F^®^, follitropin alfa; Merck Serono, Darmstadt, Germany, subcutaneous injection). The initial dose of FSH was 150–225 IU/day. The dose of gonadotropin administered was determined on the basis of the patient’s age, body mass index, and ovarian reserve parameters. Serial ultrasound examinations were used to monitor follicular and endometrial growth starting on day 6. As soon as the dominant follicle reached a diameter of 13–15 mm, daily injections of a gonadotrophin-releasing hormone (GnRH) antagonist (Cetrotide^®^ 0.25 mg/mL, Cetrorelix acetate, Merck Serono, Darmstadt, Germany) were introduced. Recombinant chorionic gonadotropin (Ovitrelle^®^, 250 µg choriogonadotropin alfa, Merck Serono, Darmstadt, Germany) was administrated when the average diameter of the three leading follicles reached 17 mm (or when at least one follicle was larger). After 34–36 h, follicular aspiration (Cook’s aspiration needle) was performed under sonographic guidance.

### 3.2. Cumulus Cell (CC) Collection

Each follicle from the patients was aspirated individually using transvaginal ultrasound guidance. The content was examined, and the cumulus oocyte complex (COC) was isolated. The CCs were enzymatically removed from the oocytes using hyaluronidase, centrifuged at 200× *g* for 5 min, and immediately subjected to further analysis. The oocytes were cultured separately.

### 3.3. Gene Expression Analysis

RNA extraction from the cumulus cells was started immediately after the collection of the cumulus cells with the addition of an RNase inhibitor. The RNA was extracted using Qiagen’s RNeasy Micro Isolation Kit. The cDNA was synthesized using an Applied Biosystems High-Capacity DNA Reverse Transcription Kit. The purity and concentration of the RNA and cDNA were determined by spectrophotometry and fluorometry (Qubit Fluorometer; Thermo Fisher Scientific, Waltham, MA, USA and BioDrop DUO; BioDro, Cambridge, UK).

The *AMH*, *AMHAMHR2*, *FSHR*, and *AR* gene expression levels were analyzed with a real-time polymerase chain reaction (RT-PCR) instrument (LightCycler 96 System; Roche, Basel, Switzerland) using TaqMan Gene Expression Assays (Applied Biosystems, Waltham, MA, USA). Normalization was performed with two different control genes, *GAPDH* and *β-actin*, as described in the literature. The RT-PCR instrument software (LightCycler 96 Software, version 1.1.0.1320; Roche, Basel, Switzerland) was used to calculate the relative gene expression values based on the delta–delta Ct method compared to the control gene values.

### 3.4. Morphological Evaluation of Oocytes, Zygotes, and Embryos

The oocytes were examined for morphological quality both before and during the ICSI procedure. ICSI was performed 3–6 h after oocyte retrieval, using only the MII oocytes isolated as part of a routine laboratory protocol that selects the MII oocytes suitable for further fertilization. The zygotes and embryos were morphologically evaluated 16–18 h and 64–66 h after fertilization, respectively.

#### 3.4.1. Morphological Evaluation of Oocytes

The oocytes that were evaluated as MII oocytes were subjected to an additional, more detailed morphological analysis, and were evaluated according to the following characteristics that provided information about the optimality of the oocytes [18]: 1. appearance of the perivitelline space (PVS), 2. presence of inclusions, 3. appearance of the first polar body (PB), 4. accumulation of organelles in the cytoplasm, 5. presence of sER disks, and 6. presence of vacuoles.

#### 3.4.2. Morphological Evaluation of Zygotes

Fertilization success was determined by the number and appearance of the pronuclei (PN), with successful fertilization defined by the presence of two visible pronuclei. A morphological assessment of the zygotes was performed based on the following characteristics [18]: 1. size of the pronuclei, 2. symmetry of the pronuclei, 3. position of the pronuclei, and 4. position of the nucleolar precursor bodies (NPBs) within the pronuclei.

After assessing the success of fertilization, the early fusion of the two gametes and the early division of the newly formed zygote were evaluated. In addition to the time of division, its regularity was also observed, as the frequency of chromosomal abnormalities is increased in zygotes that divide directly into 3 or more cells [18].

#### 3.4.3. Morphological Evaluation of Embryos

The evaluation of the embryos consisted of an assessment of the number of blastomeres formed, their morphology, and an evaluation of the percentage of fragmentation [35]. The uniformity and rate of cell division play crucial roles in assessing the morphology of an embryo. Uneven division, which is common under in vitro conditions, has a negative impact on pregnancy outcomes, while both too slow and too fast division can adversely affect implantation [36,37]. Slow division may indicate reduced implantation potential, while rapid division often leads to abnormalities [18]. A fragmentation higher than 10% is associated with an increased frequency of aneuploidy, as well as reduced embryo viability [18,37,38]. Based on all these characteristics, the oocytes, zygotes, and embryos were assigned scores from 1 to 3 (Table 2).

### 3.5. Statistical Analysis

In this study, statistical analyses were conducted to examine the effects of ovarian stimulation and modified natural cycle protocols on gene expression and reproductive cell quality in women undergoing ART. The relationships between *AMH*, *AMHAMHR2*, *FSHR*, and *AR* gene expression and the oocyte, zygote, and embryo grades were assessed using correlation coefficients. The Shapiro–Wilk test indicated non-normal distributions for *AMH*, *FSHR*, and *AR* in Group A, guiding the use of the Mann–Whitney U test, while the normally distributed *AMHAMHR2* data were analyzed with a *t*-test. Chi-square tests were used to compare the oocyte, zygote, and embryo grades across the protocols, and a multivariate regression was used to evaluate the associations between gene expression and cell quality characteristics. The analyses were performed using Statistica 14.1.0.8.

## 4. Conclusions

This study compared antagonistic COH and modified natural cycle IVF protocols in ART, examining the gene expression and morphological characteristics of oocytes, zygotes, and embryos. The key findings reveal no statistically significant differences in *AMH*, *AMHAMHR2*, *FSHR*, and *AR* expression levels between the protocols, though higher *AMH* levels were linked to successful fertilization and elevated *AMHAMHR2* to embryo transfer success, suggesting a role for these genes in specific ART stages. The morphological assessments showed similar cell quality across the protocols, with a non-significant increase in embryo transfer success under the natural cycle, possibly due to its alignment with natural hormonal rhythms. These results indicate that while both protocols are effective, gene markers like *AMH* and *AMHAMHR2* could guide protocol selection to improve outcomes.

## Figures and Tables

**Table 1:** Genes and receptors involved in AMH signaling and ovarian function. This table lists key genes and receptors involved in Anti-Müllerian Hormone (*AMH*) signaling and their roles in ovarian function. Descriptions cover functions of *AMH*, Anti-Müllerian Hormone Receptor Type 2 (*AMHR2*), Follicle-Stimulating Hormone Receptor (*FSHR*), and Androgen Receptor (*AR*), with references provided for further details on each component.

Gene	Description	Ref.
Anti-Müllerian Hormone (*AMH*)	AMH is produced by granulosa cells in ovarian follicles and acts as an indicator of ovarian reserve. It regulates folliculogenesis by preventing the excessive recruitment of primordial follicles. High levels of AMH are typically associated with a greater number of antral follicles, indicating a better ovarian reserve.	[6]
Anti-Müllerian Hormone Receptor Type 2 (*AMHAMHR2*)	AMHR2 mediates the effects of AMH on ovarian follicles. This receptor is critical for AMH function, as it influences follicle recruitment and development. The expression level of AMHR2 can provide information about follicular sensitivity to AMH, which may affect oocyte quality.	[6]
Follicle-Stimulating Hormone Receptor (*FSHR*)	FSHR is expressed on the surface of granulosa cells and is stimulated by the Follicle-Stimulating Hormone (FSH). This interaction is necessary for follicular development and estrogen production. Enhanced FSHR expression can result in improved follicular responsiveness to FSH, which is necessary for the development of multiple follicles during ovarian stimulation.	[15]
Androgen Receptor (*AR*)	ARs are involved in the action of androgens, which influences early follicular growth and development. Androgens, through ARs, can stimulate follicular recruitment and antral follicle growth. Thus, AR expression levels may influence the overall ovarian response to stimulation.	[16]

**Table 2:** Scoring of oocytes, zygotes, and embryos based on morphological assessment. This table outlines grading criteria for oocytes, zygotes, and embryos based on their morphological characteristics. Grades range from 1 to 3, with each grade reflecting number and severity of unsatisfactory morphological features. Grade 1 represents satisfactory morphology for all characteristics, while Grade 3 indicates two or more characteristics with unsatisfactory morphology in oocytes, zygotes, or embryos. For embryos, additional details on blastomere size and fragmentation are provided within each grade.

Grade	Oocyte	Zygote	Embryo
1	satisfactory morphology for all characteristics	satisfactory morphology for all characteristics	≥7 equally sized, mononuclear blastomeres, fragmentation < 20%
2	1 characteristic with unsatisfactory morphology	1 characteristic with unsatisfactory morphology	≥7 equally sized, mononuclear blastomeres, fragmentation 20–50%; 4–6 equally sized, mononuclear blastomeres, fragmentation < 20%
3	2 or more characteristics with unsatisfactory morphology	2 or more characteristics with unsatisfactory morphology	≥7 equally sized, mononuclear blastomeres, fragmentation > 50%; 4–6 equally sized, mononuclear blastomeres, fragmentation 20–50%; ≤4 equally sized, mononuclear blastomeres

**Table 3:** Chi-square test results and descriptive statistics for influence of antagonistic (A) COH and modified natural (N) protocol on oocyte, zygote, and embryo grades. This table displays mean grades and standard deviation (Mean ± SD) of oocytes, zygotes, and embryos under two protocols—antagonistic (A) COH and modified natural cycle (N). Table includes Chi^2^ and *p*-values to indicate statistical significance of differences between protocols for each grade.

	IVF Protocol	Mean ± SD	Chi^2^	*p*-Value
Oocyte grade	A	1.44 ± 0.75	0.999	0.607
	N	1.59 ± 0.86		
Zygote grade	A	1.73 ± 0.86	1.049	0.592
	N	1.91 ± 0.90		
Embryo grade	A	2.34 ± 0.81	0.749	0.600
	N	2.18 ± 0.85		

Significant difference, *p* < 0.05.

**Table 4:** The impact of antagonistic (A) COH and modified natural cycle (N) IVF protocols on individual morphological oocyte quality parameters. For each parameter, the table provides the mean values and standard deviation (Mean ± SD) for both protocols, along with the *p*-values indicating the statistical significance of any observed differences between the groups.

Oocyte Quality Parameters	A (Mean ± SD)	N (Mean ± SD)	*p*-Value
large PVS	0.10 ± 0.30	0.15 ± 0.37	0.528
inclusions in PVS	0.10 ± 0.30	0.23 ± 0.43	0.185
large 1. PB	0.03 ± 0.16	0.00 ± 0.00	0.323
accumulation of organelles in cytoplasm	0.18 ± 0.39	0.12 ± 0.33	0.502
SER discs	0.03 ± 0.16	0.00 ± 0.00	0.323
large or many small vacuole	0.05 ± 0.22	0.00 ± 0.00	0.160

Significant difference, *p* < 0.05.

**Table 5:** The impact of antagonistic (A) COH and modified natural cycle (N) IVF protocols on individual morphological zygote quality parameters. For each parameter, the table provides the mean values and standard deviation (Mean ± SD) for both protocols, along with the *p*-values indicating the statistical significance of any observed differences between the groups.

Zygote Quality Parameters	A (Mean ± SD)	N (Mean ± SD)	*p*-Value
PN different sizes	0.00 ± 0.00	0.06 ± 0.24	0.332
PN asymmetric position	0.13 ± 0.34	0.12 ± 0.33	0.907
Abnormal NPBs size and/or position	0.09 ± 0.29	0.06 ± 0.24	0.740

Significant difference (*p* < 0.05).

**Table 6:** Multivariate regression analysis of gene expression levels and individual morphological oocyte quality parameters. Table provides regression coefficients for relationship between *AMH*, *AMHAMHR2*, *FSHR*, and *AR* gene expression levels and oocyte quality parameters. Positive values indicate that higher gene expression is associated with presence of corresponding oocyte quality characteristic, while negative values suggest inverse relationship.

	Large PVS	Inclusions in PVS	Large 1. PB	Accumulation of Organelles in Cytoplasm	SER Discs	Large or Many Small Vacuole
*AMH*	0.00	0.05	0.01	−0.02	0.01	−0.02
*AMHAMHR2*	0.01	−0.05	−0.01	0.01	−0.01	0.02
*FSHR*	0.06	0.02	−0.01	−0.03	−0.01	−0.02
*AR*	−0.06	0.02	−0.01	0.01	−0.01	0.00

**Table 7:** Multivariate regression analysis of gene expression levels and individual morphological zygote quality parameters. Table provides regression coefficients for relationship between gene expression levels (*AMH*, *AMHAMHR2*, *FSHR*, and *AR*) and zygote quality. Positive values indicate that higher gene expression is associated with presence of corresponding zygote quality characteristic, while negative values suggest inverse relationship.

	PN Different Sizes	PN Asymmetric Position	Abnormal NPBs Size and/or Position
*AMH*	−0.01	0.01	−0.03
*AMHAMHR2*	0.00	0.00	0.03
*FSHR*	0.02	0.02	−0.01
*AR*	−0.01	0.01	−0.03

**Table 8:** Comparison of antagonistic (A) COH and modified natural cycle (N) IVF protocols on key assisted reproductive technology (ART) outcomes.

Outcome	IVF Protocol	YES	NO	Yes/%	*p*-Value
Fertilization	A	27	13	67.5	0.836
	N	19	7	73.1	
Embryo transfer	A	18	21	46.2	0.335
	N	16	10	61.5	

Significant difference, *p* < 0.05.

## Data Availability

The raw data supporting the conclusions of this article will be made available by the authors on request.

## Supplementary Materials

The following supporting information can be downloaded at: https://www.mdpi.com/article/10.3390/ijms252413287/s1.
