# Supplementary material for: Comparative Analysis of Controlled Ovarian Hyperstimulation and Modified Natural Cycle Protocols on Gene Expression and Quality of Oocytes, Zygotes, and Embryos in Assisted Reproductive Technology (ART)
Source: Int J Mol Sci. 2024 Dec 11;25(24):13287. doi: 10.3390/ijms252413287 (PMC11676253; doi:10.3390/ijms252413287)
Supplement: Supplementary file 1 [file ijms-25-13287-s001.zip › ijms-3330078-supplementary.pdf]

Suplementary files

Table S1. Multivariate regression analysis of gene expression levels and individual morphological oocyte quality parameters in the Antagonistic (A) COH Protocol. The table provides regression coefficients for the association between gene expression levels (AMH, AMHR2, FSHR, AR) and oocyte quality parameters under the antagonistic (A) COH protocol. Results with coefficients of zero were excluded from the table to emphasize non-zero associations between gene expression levels and quality parameters.

	**large PVS**	**inclusions in PVS**	**large 1. PB**	**accumulation of organelles in cytoplasm**	**SER discs**	**large or many small vacuole**
**AMH**	0.07	0.07	0.02	-0.09	0.02	-0.03
**AMHR2**	0.05	-0.02	-0.01	-0.05	-0.01	0.02
**FSHR**	0.08	-0.13	0.00	-0.01	0.00	-0.01
**AR**	-0.19	0.11	-0.05	0.02	-0.05	-0.01

Table S2. Multivariate regression analysis of gene expression levels and individual morphological oocyte quality parameters in the modified natural (N) cycle IVF protocol. The table provides regression coefficients for the association between gene expression levels (AMH, AMHR2, FSHR, AR) and oocyte quality parameters under the modified natural (N) cycle protocols. Results with coefficients of zero were excluded from the table to emphasize non-zero associations between gene expression levels and quality parameters.

	**large PVS**	**Inclusions in PVS**	**accumulation of organelles in cytoplasm**
**AMH**	-0.08	0.02	0.03
**AMHR2**	0.02	-0.03	0.08
**FSHR**	0.03	0.12	-0.03
**AR**	0.04	-0.05	-0.03

Table S3. Multivariate regression analysis of gene expression levels and individual morphological zygote quality parameters in the antagonistic (A) COH protocol. The table provides regression coefficients for the association between gene expression levels (AMH, AMHR2, FSHR, AR) on zygote quality parameters under the antagonistic COH (A) protocol. Results with coefficients of zero were excluded from the table to emphasize non-zero associations between gene expression levels and quality parameters.

	**PN asymmetric position**	**Abnormal NPBs size and/or position**
AMH	0.01	-0.02
AMHR2	-0.08	0.11
FSHR	0.02	-0.13
AR	0.09	0.03

Table S4. Multivariate regression analysis of gene expression levels and individual morphological zygote quality parameters in the natural (N) cycle IVF protocol. The table provides the regression coefficients for the association relationship between gene expression levels (AMH, AMHR2, FSHR, AR) and zygote quality parameters under the natural (N) cycle protocol. Results with coefficients of zero were excluded from the table to emphasize non-zero associations between gene expression levels and quality parameters.

	PN different sizes	PN asymmetric position	Abnormal NPBs size and/or position
AMH	-0.03	-0.01	0.03
AMHR2	-0.18	-0.04	-0.09
FSHR	0.12	0.05	0.02
AR	0.11	0.03	0.08

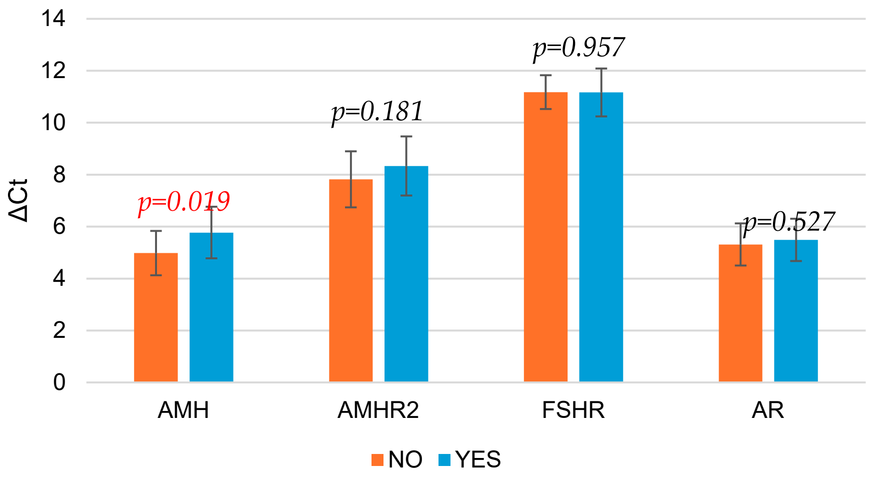

Figure S1. Comparison of gene expression levels (ΔCt values) between fertilization success outcomes in antagonistic (A) COH protocol.

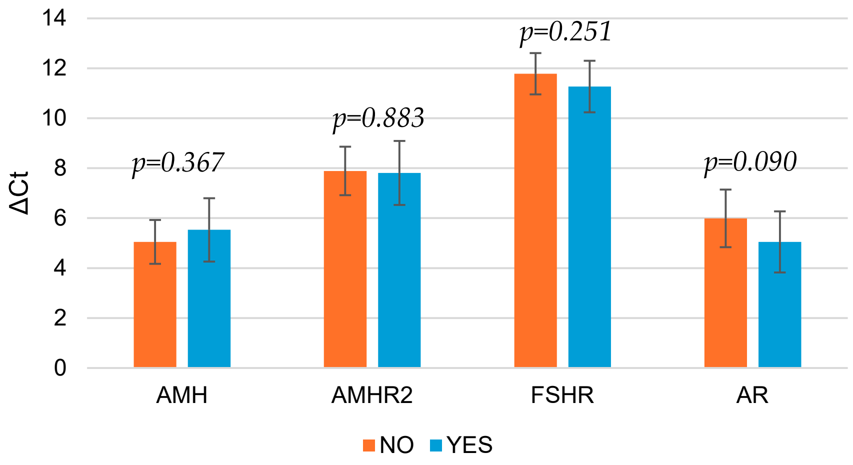

Figure S2. Comparison of gene expression levels (ΔCt values) between fertilization success outcomes in modified natural (N) cycle protocol.

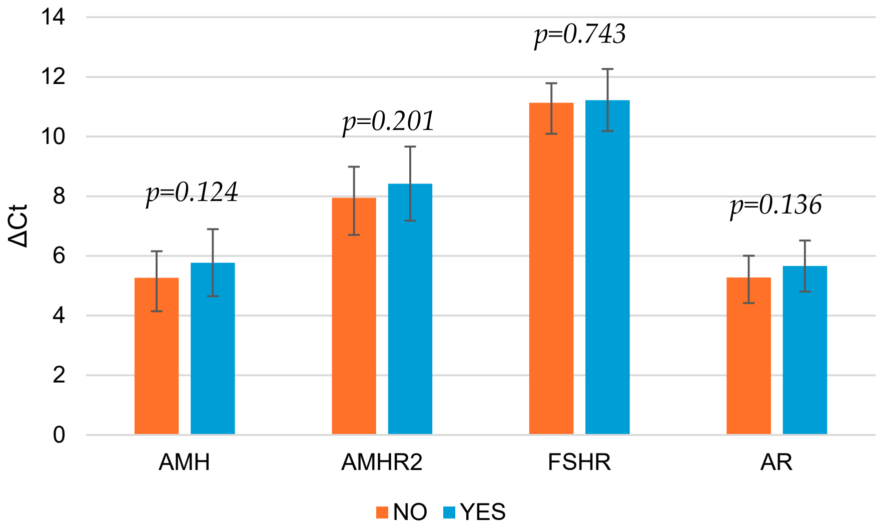

Figure S3. Comparison of gene expression levels (ΔCt values) between embryo transfer success outcomes in antagonistic (A) COH protocol.

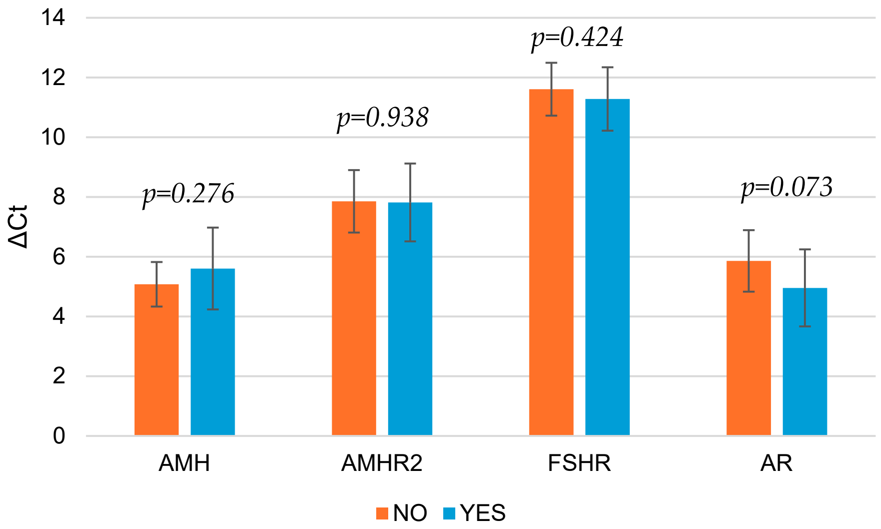

Figure S4. Comparison of Gene Expression Levels (ΔCt values) between embryo transfer success outcomes in modified natural (N) cycle protocol.
